# Educational Expectations and Academic Persistence among Rural Adolescents: The Protective Role of High Self-Esteem

**DOI:** 10.3390/bs14100888

**Published:** 2024-10-01

**Authors:** Feng Zhang, Xiaodan Xu, Wei Peng, Cheng Guo

**Affiliations:** 1 Institute of Applied Psychology, Psychological Research and Counseling Center, Southwest Jiaotong University, Chengdu 610031, China; zhangfpsy@swjtu.edu.cn; 2 School of Arts and Communication, Beijing Normal University, Beijing 100875, China; 3 School of Economics and Social Welfare, Zhejiang Shuren University, Hangzhou 310015, China; 601476@zjsru.edu.cn; 4 Mental Health Education Center of Student, Qinghai University, Xining 810016, China; 2021990082@qhu.edu.cn

**Keywords:** educational expectations, persistence, self-esteem, rural family

## Abstract

Rural adolescents are at higher risk of reduced academic persistence due to socioeconomic barriers. Educational expectations are theoretically viewed as important for adolescents’ learning behaviors, and cross-sectional research has supported this assumption. However, few longitudinal studies have investigated the influence of educational expectations on adolescents’ academic persistence. In addition, research has not clearly identified whether self-esteem moderates this link among adolescents who experience greater economic risk. Using data from two time points (i.e., six months apart), this study aims to provide a more complete understanding of whether, and under what conditions, rural adolescents’ educational expectations influence academic persistence. The participants consist of 631 adolescents (*M*_age_ = 13.34 years at T1), and all the adolescents are from families with rural household registrations. The results show that the interaction term of educational expectations and self-esteem significantly predicts academic persistence. Specifically, after controlling for baseline academic persistence, educational expectations positively predict later academic persistence for rural adolescents with lower self-esteem, and educational expectations do not significantly predict later academic persistence for those with higher self-esteem. This study reveals the protective role of self-esteem in rural adolescents. High self-esteem benefits rural adolescents by protecting them from the effects of lower educational expectations on academic persistence. This finding also emphasizes the importance of developing self-esteem interventions for rural adolescents with low educational expectations to prevent them from experiencing weaker academic persistence.

## 1. Introduction

China has made tremendous efforts to reduce the widening of the rural–urban income gap [1,2]. Partly because of rural–urban income inequality, residents of rural families face a variety of disadvantages in terms of social public services, such as education, health care, and housing [3]. Specifically, rural residents still lag behind urban residents in terms of income, education, and occupation, making their socioeconomic status (SES) extremely low in modern Chinese society [4]. This is especially true for rural residents in the western provinces of China because of unbalanced economic development [3]. As such, adolescents from rural families may encounter greater socioeconomic risk, which may be related to poor mental health development [5] and academic behaviors [6]. Continuous psychological assistance is needed to promote rural adolescents’ long-term development. Academic persistence is regarded as a behavioral outcome of self-regulation [7] and involves adolescents’ enduring participation and effort in learning activities in this study. According to previous studies [8,9], academic persistence is a noncognitive factor that is indispensable for adolescents’ ultimate success. A greater degree of perseverance in learning may be correlated with better educational outcomes, thus leading to a better future life [10,11]. Considering its benefits in advancing positive outcomes, it is important to identify the factors associated with academic persistence in adolescents from rural families to seek opportunities to maintain and enhance academic persistence. More importantly, adolescence is a key period for the development of academic persistence. Related evidence has suggested that adolescents’ behavioral engagement declines as they enter middle school, partly because of increased academic challenges and difficulties [12]. A decrease in engagement may lead to more problem behaviors [13]. Given that academic persistence is a concept related to behavioral engagement, it is possible that academic persistence is also at risk of declining in adolescence, a phenomenon which may not be conducive to positive developmental outcomes for adolescents. Thus, investigating the factors that contribute to academic persistence is important for adolescents.

Expectancy–value theory elaborates that both the expectancies for success and the values of the task can guide individuals’ task-related behaviors, persistence, and choices [14]. In particular, expectancies for success refer to individuals’ beliefs about how well they will perform with respect to immediate or future goals and tasks. As such, the educational expectations on which this study focuses are analogous to the expectancy beliefs this theory elaborates. Thus, on the basis of the expectancy–value theory, educational expectations have long been hypothesized to be related to adolescents’ academic achievement, learning-related behaviors, and persistence. The correlations between educational expectations and academic achievement have been well established in empirical studies [15,16]. However, less attention has been given to the relationships between educational expectations and learning-related behaviors (e.g., academic persistence). Moreover, owing to the multiple risks faced by rural adolescents, their educational expectations may decrease [17], possibly leading to a decrease in their persistence with academic tasks. According to the resilience model [18], some factors can protect individuals from the negative impact of risk environments. These individuals are considered resilient, and these factors are considered resilience resources or protectors of individuals’ positive developmental outcomes in the face of risk. Self-esteem may act as a resilience factor that accounts for why some adolescents are more perseverant than others in the context of low educational expectations in rural areas. Research focused on rural adolescents is of practical value, as it can provide evidence on how to provide psychological assistance to effectively promote their development, especially in the education field. Therefore, this study targets adolescents from rural families to fully understand the associations among educational expectations, self-esteem, and academic persistence.

### 1.1. Educational Expectations and Academic Persistence in the Context of Rural Families

Educational expectations are based on the highest educational level that adolescents believe they will attain [19,20]. Educational expectations are associated with positive learning behaviors and better academic performance. More specifically, cross-sectional studies have shown that educational expectations correlate with cognitive performance and academic achievement [21,22], and longitudinal research has shown that adolescents’ educational expectations predict their academic attainment in adulthood [23].

According to situated expectancy–value theory [14,24], adolescents’ expectations for educational attainment may motivate and guide their academic-related behaviors. A few studies have directly shown a positive correlation between educational expectations and academic persistence [25]. Some concepts that are closely related to academic persistence have provided support for this study. For example, adolescents with higher educational expectations are more engaged in their learning [26] and exhibit higher levels of learning motivation [19]. In addition, adolescents with higher educational expectations demonstrate more positive learning behaviors [27]. Both learning engagement and motivation are closely associated with academic persistence [28]. In this way, educational expectations may be correlated with adolescents’ academic persistence. However, the relationship between educational expectations and academic persistence has received less attention. This study explicitly addresses this issue and explores the short-term relationship between educational expectations and academic persistence.

In addition, rural adolescents’ educational expectations are generally lower [29], largely due to sociostructural barriers, such as lower family SES and relatively poor rural environments. For example, low-income adolescents face greater risk in their learning and, thus, they may lower their educational expectations [17]. Moreover, although adolescents may initially possess higher educational expectations, their educational expectations can be reduced by the limited family environment over time [30]. Therefore, it is necessary to explore whether self-esteem can mitigate the relationship between educational expectations and academic persistence for adolescents from rural families.

### 1.2. Potential Moderating Role of Self-Esteem

Self-esteem depends on individuals’ evaluations of their total worthiness [31]. This notion could also be further divided into general or global and domain-specific self-esteem, a classification which can be applied to various contexts, such as home and school self-esteem [32,33]. The present study focuses on global self-esteem, which does not distinguish among specific contexts but rather focuses on an overall evaluation of worthiness. Adolescents with high self-esteem possess the ability to cope with negative events, and, thus, optimize their psychological and behavioral development. Previous research has shown that self-esteem is generally associated with adolescents’ positive outcomes [34,35], such as greater academic engagement [36] and enhanced persistence after failure [37]. In addition, self-esteem is considered a resilience resource for at-risk adolescents to regulate negative influences [38,39], and evidence that high self-esteem prevents individuals from experiencing the negative effects of stress-related factors on distress has been documented extensively [40,41,42,43]. This empirical evidence, together with the resilience model above, clearly supports the emotion regulation model of self-esteem [44] and shows that high self-esteem helps individuals counteract the negative effects of stressors and maintain psychological well-being. As such, recent research has witnessed the increase in evidence that high self-esteem relieves adverse emotional reactions to negative environments, events, and cognitions. However, a lack of evidence has revealed the buffering effects of self-esteem on behavioral outcomes in at-risk individuals. This study aims to fill this gap by investigating whether self-esteem alleviates the effects of low educational expectations on the academic persistence of rural adolescents.

Both direct and related evidence has revealed positive correlations between self-esteem and academic persistence. Direct experimental evidence has demonstrated the causal link between self-esteem and persistence in difficult tasks [37]. Individuals with high self-esteem are more likely to persist when faced with difficult tasks [45] and are also more inclined to set higher goals in the face of challenging tasks [46]. Some indirect evidence has also revealed positive correlations between self-esteem and variables related to adolescents’ academic persistence, such as academic engagement [47] and grit [48,49]. According to the self-enhancement theory [44,50], all individuals have fundamental motives to enhance and restore their favorable self-views despite using different strategies. Adolescents with high self-esteem are more likely to persevere because they are motivated to enhance their positive self-views. Adolescents with low self-esteem might tend to avoid failure because of a desire to protect themselves from the loss of their already flawed selves by refusing to take on challenges. In this way, it is reasonable to assume that self-esteem positively predicts adolescents’ persistence in academic domains. In particular, one empirical study revealed the buffering role of self-esteem in the link between persistence and negative social evaluations [51]. After receiving a negative social evaluation, individuals with low self-esteem focus on their own shortcomings and inferiority, and this frustrating self-view can make it easier for them to give up, demonstrating lower levels of persistence than individuals with high self-esteem.

Taken together, based on the evidence that self-esteem regulates negative effects and considering that self-esteem acts as a resilience resource for at-economic-risk adolescents [18], this study assumes that self-esteem could also mitigate the effects of low educational expectations on the academic persistence of rural adolescents. The moderating effects might work as follows. Primarily, previous research has shown that lower educational expectations are often accompanied by lower self-efficacy for academics [21,52], and this deficient self-view might weaken the persistence of adolescents with low educational expectations. High self-esteem supplements feelings of incompetence and maintains adolescents’ academic persistence; in other words, academic persistence is less influenced by low educational expectations when adolescents possess high self-esteem. Moreover, as previous research has indicated [53], self-esteem is closely related to positive cognitions (i.e., the cognitive accessibility of strengths). Adolescents with high self-esteem have greater access to strengths, which can help them demonstrate high persistence when facing adverse factors. In contrast, adolescents with low self-esteem are more likely to associate their failure experiences and weaknesses, leading to their avoidance tendencies. In this way, without obvious explicit goals (e.g., pursuing higher education), adolescents with low self-esteem have no motives to persist in relation to academic-related tasks, demonstrating low academic persistence. Therefore, the relationship between educational expectations and academic persistence varies according to the degree of self-esteem.

### 1.3. The Current Study

This study echoes the expectancy–value theory [14,24] to investigate the longitudinal relationship between educational expectations and academic persistence among adolescents from rural families. Moreover, based on the resilience model of self-esteem for adolescents at economic risk [18], this study tests whether self-esteem moderates the relationship between educational expectations and academic persistence. This study speculates that high self-esteem would protect rural adolescents’ academic persistence from the adverse effects of low educational expectations. In addition, to fully understand the associations among educational expectations, self-esteem, and academic persistence, the economic context (e.g., rural families whose economic conditions are at greater risk) in which adolescents live matters, as education is an important pathway through which to seek a better occupation in urban cities, thus improving the prospects of relatively poor families. However, less evidence has explicitly explored the associations among educational expectations, self-esteem, and academic persistence in rural adolescents. Therefore, this study focuses on rural adolescents to explore the above associations. Specifically, self-esteem is hypothesized to moderate the relationship between educational expectations and academic persistence; when rural adolescents have higher self-esteem, low educational expectations do not affect their academic persistence.

## 2. Materials and Methods

### 2.1. Participants

All participants were recruited from a middle school located in a small town in a county, which is located in the northern part of Sichuan Province in China. Specifically, the students who attended this town school in the county were all from local villages and small towns, and the county was once a national-level poverty-stricken county with a relatively low level of regional economic development. Thus, the students came from families with rural and urban household registrations (Hukou), something which was convenient to obtain the target sample of this study.

At time 1 (T1), a total of 631 seventh- to eighth- grade adolescents from families with rural Hukou participated in this study (*M*_age_ = 13.34 years, *SD* = 0.74, 55.20% boys). Six months later, at time 2 (T2), the adolescents were invited once again, and 569 data point were collected (*M*_age_ = 13.83 years, *SD* = 0.68 at T2, and 56.10% boys). From T1 to T2, sixty-two students (approximately 10%) dropped out of the study due to being transferred to a new school or due to incomplete or partially completed questionnaires.

All of the participants’ home addresses were located in the surrounding rural areas, far from their attended school in the town. The majority of the adolescents were residential students who went home on weekends or holidays (approximately 79.96%). The remaining adolescents lived with their parents in rented houses around the town. Moreover, another two pieces of demographic information on all the participating adolescents were collected, including parental status and whether they were only-children in their families. Specifically, most adolescents (*n* = 509, 89.46%) were from two-parent families, while a small proportion of adolescents (*n* = 60, 10.55%) were from single-parent families (i.e., divorced parents or, unfortunately, one of the parents had passed away). In addition, most adolescents reported that they were from non-only-child families (85.60%), and the remaining adolescents (14.40%) were from only-child families.

Furthermore, information on the parental education level is shown in Table 1. Most of the adolescents’ paternal educational levels were below associate degree (98.60%), and most adolescents’ maternal educational levels were also below associate degree (97.80%), indicating relatively low parental educational levels.

### 2.2. Procedure

This was a longitudinal study, and the data were obtained from both the adolescents’ and their parents’ reports. Prior to the data collection, the investigation protocol was approved by the local educational department and the administration of the school. All the participants were informed that their participation was voluntary and that they could withdraw at any time. Specifically, the data were measured twice from the adolescents’ reports. 

At the beginning of the spring semester, 631 valid samples from families with rural Hukou who provided informed consent participated in this study. The questionnaires for the parents were sealed in envelopes. Each adolescent was asked to take the envelope home on Friday and return it to the investigators the next Monday. Fathers or mothers who were the primary caregivers of the target adolescents were asked to provide information on both paternal and maternal highest educational levels. In addition, the adolescents completed questionnaires on their demographic information, educational expectations, self-esteem, and academic persistence in their classrooms. Approximately six months later, at T2, the adolescents were invited again to report on their academic persistence. In particular, the span of one semester provided this study with an opportunity to follow changes in academic persistence, as adolescents inevitably encounter learning challenges and difficulties as they continue to gain new knowledge across the semester. In this way, this study can present the changes in academic persistence throughout one semester.

The final 569 adolescents reported valid data at two time points and were thus used for subsequent data analyses. After the completion of the investigation, each adolescent received a gift (approximately $1.5) as appreciation for his or her participation.

### 2.3. Measures

#### 2.3.1. Educational Expectations

Educational expectations measure the educational level that adolescents expect themselves to achieve in the future. At T1, adolescents answered one item about how far they expected they would go to school on an eight-point scale (1 = junior high school, 2 = technical secondary school graduate, 3 = vocational high school completed, 4 = senior high school completed, 5 = associate degree completed, 6 = bachelor’s degree obtained, 7 = master’s degree obtained, and 8 = doctoral degree obtained). Higher scores represent higher levels of educational expectations at T1.

#### 2.3.2. Self-Esteem

The adolescents’ self-esteem was measured by using the ten-item Rosenberg self-esteem scale [31] (e.g., “On the whole, I am satisfied with myself”). The Rosenberg self-esteem scale is a well-developed and widely used tool for assessing individuals’ global self-esteem. A previous study demonstrated the reliability of this scale across nations [54], and this scale has been successfully used among Chinese adolescents [55]. At T1, the adolescents reported how strongly they agreed with each item on a scale ranging from 1 (strongly disagree) to 4 (strongly agree). After the items were reverse scored, the mean scores of all 10 items were calculated, with higher scores reflecting higher levels of self-esteem at T1. The Cronbach’s alpha was 0.84 in the present study.

#### 2.3.3. Academic Persistence

At T1 and T2, academic persistence was measured by using the five items adopted from the OECD Programme for International Student Assessment (PISA) surveys [56]. PISA surveys collect data on 15-year-old adolescents in participating countries (e.g., China) every three years. The items concerning persistence were first added in 2012 and they demonstrated acceptable reliability for Chinese students. In this study, minor content (i.e., in my daily learning and study) was added to each original item to ensure the measurement of adherence in the learning field. Specifically, adolescents were rated on a scale ranging from 1 (not at all like me) to 5 (very much like me) (e.g., in my daily learning and study, “When confronted with a problem, I give up easily”; “I continue working on tasks until everything is perfect”). Two items were reverse scored, and the mean scores of all the items were subsequently calculated, with higher scores indicating higher levels of academic persistence. The Cronbach’s alpha was 0.89 at T1 and 0.92 at T2 in the present study.

### 2.4. Data Analyses

All the data analyses were conducted via SPSS 25.0 and the SPSS PROCESS macro. First, the descriptive statistics were analyzed, and the means, standard deviations, and correlations of the variables are shown. Second, the SPSS PROCESS macro was used to test the moderating effect of adolescent T1 self-esteem on the relationship between T1 educational expectations and T2 academic persistence. Specifically, MODEL 1 in the PROCESS macro was carried out with one thousand bootstrapping resamples to generate 95% confidence intervals; the moderating model was considered effective when the interaction item was significant and the 1000 bootstrapped 95% confidence intervals did not include zero. The adolescents’ gender, age, parental educational level, and academic persistence at T1 were controlled for in the subsequent analyses. Gender was transformed into a dummy variable (boy = 0, girl = 1) and age was z-transformed. All the other variables were standardized to z scores. Finally, a simple slope diagram was generated when the moderating effect was significant.

In particular, previous studies have revealed gender differences in school engagement [57], a concept related to academic persistence. Thus, to provide a fuller understanding of the gender differences in the main variables for the current study, an independent sample *t* test was conducted. The results revealed nonsignificant differences in T1 educational expectations (*M*_boys_ = 6.13, *SD* = 1.40; *M*_girls_ = 6.11, *SD* = 1.23; *t*_(629)_ = 0.15, *p* = 0.884) and T1 self-esteem (*M*_boys_ = 2.74, *SD* = 0.58; *M*_girls_ = 2.66, *SD* = 0.60; *t*_(629)_ = 1.89, *p* = 0.059) between boys and girls. Moreover, significant differences were also shown. Boys exhibited higher scores for both T1 and T2 academic persistence (for T1: *M*_boys_ = 2.86, *SD* = 1.00; *M*_girls_ = 2.54, *SD* = 0.87; *t*_(629)_ = 4.31, *p* < 0.001; for T2: *M*_boys_ = 3.02, *SD* = 0.89; *M*_girls_ = 2.66, *SD* = 0.84; *t*_(567)_ = 4.86, *p* < 0.001).

## 3. Results

### 3.1. Descriptive Statistics and Correlations among the Main Variables

The means, standardized deviations, and correlations among the main study variables are shown in Table 2. The main results show that both T1 educational expectations (*r* = 0.15, *p* < 0.001) and T1 self-esteem (*r* = 0.32, *p* < 0.001) were significantly related to T2 academic persistence. T1 self-esteem was positively related to T1 educational expectations (*r* = 0.18, *p* < 0.001).

### 3.2. Moderating Effects of Adolescent Self-Esteem

As shown in Table 3, after controlling for gender, age, parental educational level, and academic persistence at T1, the results indicate that T1 educational expectations did not significantly predict adolescents’ T2 academic persistence (*β* = 0.07, *p* = 0.08). T1 self-esteem significantly and positively predicted T2 academic persistence (*β* = 0.15, *p* < 0.001). In addition, T1 self-esteem moderated the relationship between T1 educational expectations and T2 academic persistence (*β* = 0.10, *p* = 0.008).

**Figure 1:**
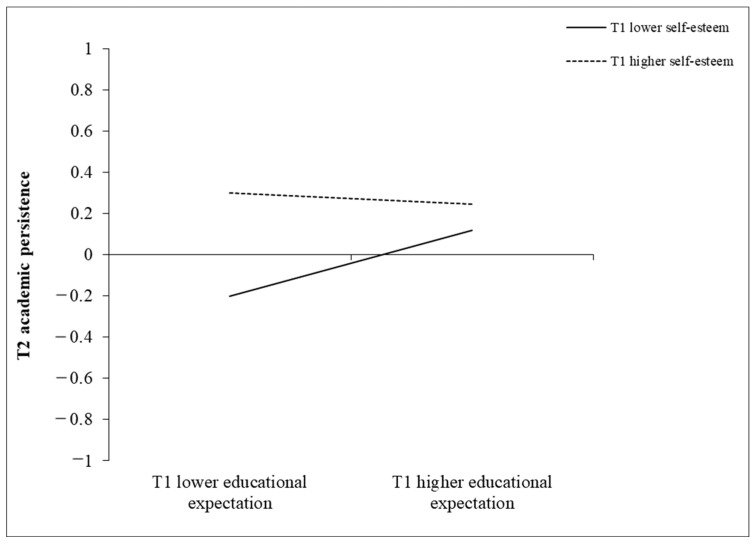
The moderating role of self-esteem. (Note. The solid line indicates a significant relationship at a *p* value lower than 0.05, while the dotted line indicates a nonsignificant relationship with a *p* value greater than 0.05).

The simple slope test for the two-way interactions is shown in Figure 1. The results indicate that, for adolescents with lower levels of T1 self-esteem, T1 educational expectations positively predicted T2 academic persistence (simple slope = 0.17, *t* = 3.80, *p* < 0.001, 95% *CI* = [0.07, 0.28]), and, when adolescents possessed higher levels of T1 self-esteem, T1 educational expectations were not significantly related to T2 academic persistence (simple slope = −0.03, *t* = −0.58, *p* = 0.561, 95% *CI* = [−0.14, 0.08]).

## 4. Discussion

When confronted with challenging academic tasks, some adolescents are perseverant and put effort into difficult tasks, but others give up easily and lose the chance to succeed in such tasks. Educational expectations might be a possible explanation for adolescents’ academic persistence, and cross-sectional research has, thus far, supported this view. However, less is known about whether educational expectations predict later academic persistence or whether self-esteem moderates this relationship. This study explicitly addresses this issue by targeting adolescents in rural areas. As a result, a moderating role of self-esteem was found. Specifically, for rural adolescents with high self-esteem, educational expectations had less of an impact on later academic persistence; for rural adolescents with low self-esteem, educational expectations had a significant impact on later academic persistence. As such, rural adolescents with lower self-esteem might benefit more from educational expectations in terms of their development of academic persistence over time. In other words, high self-esteem protects rural adolescents’ academic persistence from the negative effects of low educational expectations.

Based on the expectancy–value theory [14,24], educational expectations are supposed to be positively correlated with adolescents’ academic persistence. Related research using cross-sectional data supports this assumption [19]; the greater the educational expectation, the greater the behavioral efforts toward academic achievements [25]. Our findings are consistent with this theory and extend related empirical evidence, showing that educational expectations are positively correlated not only with current but also with future academic persistence in a sample of rural adolescents. More importantly, as indicated by the results of the regression model, when self-esteem was added, self-esteem could directly predict an increase in academic persistence. Meanwhile, educational expectations positively predicted an increase in academic persistence at lower levels of self-esteem. As such, the effect of educational expectations on academic persistence were found to depend on the self-esteem of rural adolescents. In particular, this study reveals that self-esteem complements educational expectations that, together, benefit future academic persistence in the context of rural families. When educational expectations are low, high self-esteem can protect rural adolescents from the effects of such low educational expectations, maintaining greater future academic persistence. When self-esteem is low, educational expectations can positively predict rural adolescents’ future academic persistence.

According to previous research [58], a high-economic-risk environment could impair adolescents’ expectations with respect to future educational attainment. For example, adolescents with low family SES can initially expect to attain a high level of education [59,60], but they may gradually lower their educational expectations over time [17], partly because of limited environmental resources. In this way, rural adolescents may experience the adverse effect of low educational expectations, which do not motivate them to persevere. Fortunately, the protective role of high self-esteem for adolescents in rural areas is shown in this study, with rural adolescents with high self-esteem found to exhibit stronger academic persistence six months after the initial survey, irrespective of educational expectations. Previous research has described the emotion regulation model of self-esteem [40,44,61], and, combined with the findings of this study, self-esteem is beyond its ability to regulate negative emotions in response to negative stressors. Self-esteem could not only buffer persistence after receiving negative social evaluations [51], but also maintain academic persistence in the face of low educational expectations. Thus, the protective role of high self-esteem revealed in our study is consistent with and extends previous evidence by uncovering the regulatory function of self-esteem in behavioral development.

In addition, in line with the resilience model [18,39], high self-esteem could act as a resilience resource to help rural adolescents maintain their academic persistence when they have low educational expectations. Low educational expectations are associated with incompetence and lower self-efficacy [21,52]. As such, rural adolescents with low educational expectations are at greater risk of deficiency or incompetence, which makes them unable to persevere in academic tasks. Adolescents with high self-esteem may possess greater perceived competence than adolescents with low self-esteem. Specifically, previous research has focused on adolescents with greater economic risk and found that those adolescents with high self-esteem are more confident in their own ability [50] and that adolescents with low self-esteem exhibit lower self-efficacy [62]. In addition to differences in competence, adolescents with high and low self-esteem might also differ in social support resources [63], and effective social support (e.g., assistance from parents) may promote individuals’ perseverance [64]. Thus, when confronted with low educational expectations, rural adolescents with low self-esteem are at double risk of incompetence and less effective support, leading them to demonstrate the lowest academic persistence. In contrast, low educational expectations do not affect the academic persistence of rural adolescents with high self-esteem. Because these adolescents have more affluent psychological and external resources (e.g., competence, efficacy, and social support), these resilient resources could directly maintain rural adolescents’ academic persistence, and educational expectations have less of an impact herein.

Finally, this study reveals that, on average, boys had higher levels of academic persistence than girls did. Previous studies have shown that boys have lower levels of school engagement (e.g., school compliance, participation in extracurricular activities and school identification) than girls do [57]. Our findings differ from this previous evidence and add new evidence with respect to the gender differences in academic persistence in the rural familial context. This is probably because, in traditional Chinese society, sons are more valued, respected, and supported than daughters are, a phenomenon which may be especially true in rural families [65]. In fact, well-developed family relationships, such as parental encouragement and autonomy support, benefit their children’s persistence [66,67]. As such, rural boys might receive more psychological resources that can support their perseverance in the face of academic risk. Conversely, rural girls are at a disadvantage with respect to accessing the necessary support resources, something which may unintentionally prevent them from persevering through difficult academic tasks. Nevertheless, this possible explanation for the gender difference in academic persistence in the rural familial context requires further exploration in future research.

## 5. Limitations and Practical Implications

Several limitations of this study should be addressed. First, all the target adolescents were from families with rural household registrations and attended a small township school. Moreover, almost all parents’ educational levels were relatively low (presented in Table 1), indicating that the adolescents were all coming from economically disadvantaged families. More objective information regarding economic conditions (e.g., family assets, healthcare welfare, and housing) should be sought by future research to provide a more comprehensive picture of the economic risk levels faced by these rural adolescents. Second, a previous study measured persistence through difficult and challenging tasks [64]; future research could also measure persistence through similar academic tasks to collect multisource data to provide a more complete understanding of academic persistence. Third, although the present study reveals positive effects of self-esteem on rural adolescents’ academic persistence, the negative effects of high self-esteem, as suggested by previous studies, should also be highlighted. Specifically, self-esteem is generally associated with a variety of important life outcomes [35,68]. Despite the benefits of high self-esteem, some research has revealed negative effects of high self-esteem on academic-related behaviors. For example, evidence has revealed that adolescents with high self-esteem may attempt to protect their positive self-evaluations by putting less effort into tasks [37,45,69]. Therefore, future research could focus on the boundary conditions under which high self-esteem promotes individuals’ positive development.

Despite these limitations, these findings are important for the development of educational interventions aimed at promoting academic persistence in rural adolescents. Adolescents in rural areas are regarded as members of an “at-risk” group that needs interventions to facilitate positive outcomes [70]. The two basic sets of self-perceptions on which this study focused, i.e., the values and goals individuals want to achieve (expectations of educational attainment in the future) and the overall evaluations of who they are (self-esteem), are key motivators of learning-related actions. Thus, cultivation of these two facets of the “self” by rural adolescents might be helpful for their development of academic persistence. In addition, although self-esteem is generally regarded as a trait variable that is difficult to change, it can be promoted through properly designed intervention programs [68]. Overall, this study reveals that, for Chinese rural families, educational expectations and self-esteem have complementary effects on academic persistence. When rural adolescents’ self-esteem is low, high educational expectations could help them persevere more academically; when rural adolescents’ educational expectations are low, high self-esteem could protect them from the effects of low educational expectations on academic persistence. In this way, both educational expectations and self-esteem are important for predicting academic persistence. Therefore, the findings can help develop interventions aimed at rural adolescents. Notably, the promotion of not only expected future educational levels but also self-esteem could allow rural adolescents to demonstrate greater academic persistence, which ultimately increases the likelihood of their success in the future.

## Figures and Tables

**Table 1:** Frequency statistics of parents’ educational level.

Educational Level	Fathers’		Mothers’	
	Frequency	Proportion	Frequency	Proportion
1. Primary school and primary school not completed	214	33.90%	219	34.70%
2. Junior high school completed	297	47.10%	281	44.50%
3. Technical secondary school completed	21	3.30%	20	3.20%
4. Vocational high school completed	15	2.40%	17	2.70%
5. Senior high school completed	70	11.10%	80	12.70%
6. Associate degree completed	5	0.80%	8	1.30%
7. Bachelor’s degree obtained	9	1.40%	6	1.00%
8. Master’s degree obtained	0	0.00%	0	0.00%
9. Doctoral degree obtained	0	0.00%	0	0.00%

**Table 2:** Descriptive statistics and correlations for all variables.

Variables	*M*	*SD*	1	2	3	4	5	6	7
1. Gender	0.45	0.50	—						
2. T1 age	13.34	0.74	−0.09 *	—					
3. T1 father’s educational level	2.18	1.39	−0.01	−0.11 **	—				
4. T1 mother’s educational level	2.22	1.42	−0.001	−0.07	0.42 ***	—			
5. T1 academic persistence	2.72	0.96	−0.17 ***	−0.03	0.05	−0.02	—		
6. T1 educational expectations	6.13	1.33	−0.01	−0.01	0.14 **	0.08	0.14 ***	—	
7. T1 self-esteem	2.70	0.59	−0.08	0.08	0.03	0.09 *	0.43 ***	0.18 ***	—
8. T2 academic persistence	2.86	0.89	−0.20 ***	−0.003	−0.02	0.01	0.46 ***	0.15 ***	0.32 ***

Note. Gender is a dummy variable (boy = 0, girl = 1); *** *p* < 0.001, ** *p* < 0,01 and * *p* < 0.05.

**Table 3:** Moderation model for adolescents’ academic persistence at T2.

Predictors	T2 Academic Persistence			*R* ^2^	*F*
	*β*	*SE*	95%*CI*		
T1 educational expectations	0.07	0.04	[−0.01, 0.15]	0.27	25.80 ***
T1 self-esteem	0.15 ***	0.04	[0.07, 0.23]		
T1 educational expectations × self-esteem	−0.10 **	0.04	[−0.17, −0.03]		
Gender	−0.27 ***	0.07	[−0.41, −0.12]		
T1 age	−0.01	0.04	[−0.08, 0.06]		
T1 father’s educational level	−0.07	0.04	[−0.15, 0.01]		
T1 mother’s educational level	0.04	0.04	[−0.04, 0.12]		
T1 academic persistence	0.39 ***	0.04	[0.31, 0.47]		

Note. Gender was a dummy variable; all the other continuous variables were transformed into z scores. *** *p* < 0.001, ** *p* < 0.01.

## Data Availability

The data that support the findings of this study are available upon request from the authors.
